# Provider-agency fit in substance abuse treatment organizations: implications for learning climate, morale, and evidence-based practice implementation

**DOI:** 10.1186/s13104-015-1110-3

**Published:** 2015-05-12

**Authors:** Alex T Ramsey, Carissa van den Berk-Clark, David A Patterson Silver Wolf (Adelv unegv Waya)

**Affiliations:** Washington University Brown School of Social Work, Campus Box 1196, One Brookings Drive, St. Louis, MO 63130 USA; Department of Psychiatry, Washington University School of Medicine, Medical Box 8134, St. Louis, Missouri 63110 USA; Veterans Health Administration Research and Development, 501 N. Grand Blvd., St. Louis Healthcare System, St. Louis, Missouri 63103 USA; Department of Family and Community Medicine, Saint Louis University, St. Louis, Missouri USA

**Keywords:** Evidence-based practice implementation, Practitioner attitudes, Organizational climate, Person-environment fit, Substance abuse treatment agencies, Addiction services

## Abstract

**Background:**

Substance abuse agencies have been slow to adopt and implement evidence-based practices (EBPs), due in part to poor provider morale and organizational climates that are not conducive to successful learning and integration of these practices. Person-organization fit theory suggests that alignment, or fit, between provider- and agency-level characteristics regarding the implementation of EBPs may influence provider morale and organizational learning climate and, thus, implementation success. The current study hypothesized that discrepancies, or lack of fit, between provider- and agency-level contextual factors would negatively predict provider morale and organizational learning climate, outcomes shown to be associated with successful EBP implementation.

**Methods:**

Direct service providers (n = 120) from four substance abuse treatment agencies responded to a survey involving provider morale, organizational learning climate, agency expectations for EBP use, agency resources for EBP use, and provider attitudes towards EBP use. Difference scores between combinations of provider- and agency-level factors were computed to model provider-agency fit. Quadratic regression analyses were conducted to more adequately and comprehensively model the level of the dependent variables across the entire “fit continuum”.

**Results:**

Discrepancies, or misfit, between agency expectations and provider attitudes and between agency resources and provider attitudes were associated with poorer provider morale and weaker organizational learning climate. For all hypotheses, the curvilinear model of provider-agency discrepancies significantly predicted provider morale and organizational learning climate, indicating that both directions of misfit (provider factors more favorable than agency factors, and vice-versa) were detrimental to morale and climate. However, outcomes were most negative when providers viewed EBPs favorably, but perceived that agency expectations and resources were less supportive of EBP use.

**Conclusions:**

The current research benefits from a strong theoretical framework, consistent findings, and significant practical implications for substance abuse treatment agencies. Comprehensive attempts to strengthen outcomes related to EBP implementation must consider both provider- and agency-level characteristics regarding EBP use. Organizational efforts to more closely align provider attitudes and agency priorities will likely constitute a key strategy in fostering the implementation of EBPs in substance abuse treatment organizations.

## Background

It has been well established that behavioral health service providers, particularly those specializing in substance abuse treatment, commonly fail to utilize evidence-based practices (EBP) [1-3]. These practices refer to empirically-supported manualized therapies, treatments, and interventions with specific guidelines or components outlined in a manual that are to be followed in a structured or predetermined way. The factors contributing to the effective implementation of EBPs in addiction treatment programs are complex and multifaceted. Paramount among these issues are infrastructure and resource constraints, characterized by a lack of full-time providers, limited education and training among providers, inadequate equipment and decision support, inefficient intake processes due to reporting requirements, frequent reorganization of agencies, and disturbingly high turnover rates among counselors and directors [4]. Further, many existing treatment models are based on folk wisdom from those in recovery [5] which has likely impeded providers’ readiness and motivation to adopt EBPs [6]. However, while the numerous barriers to use of EBPs reflect a grim picture of addiction services, recent research has begun to identify factors that are indicative of successful implementation in substance abuse treatment agencies.

### Outcomes indicating successful evidence-based practice implementation

#### Organizational learning climate

To date, much health services research has examined the profiles of agencies most likely to adopt and successfully implement empirically supported interventions [6,7]. One key profile indicator includes organizational climate, which reflects an agency’s capacity to integrate innovations into practice [8-10]. Research indicates that organizational climate may be particularly important during the active implementation and maintenance stages of the implementation process, ensuring that adequate resources (e.g., financial, equipment, personnel) are in place to support continued integration of the EBP [10]. Further, organizational research has demonstrated that learning climates which encourage team learning processes are more likely to experience implementation success [11-13]. In fact, Pisano et al. [12] found that successful agencies adopting new practices exhibited significantly different team learning characteristics as compared to less successful teams. While much research has examined organizational climate more broadly, less attention has been given to organizational learning processes and capacity to learn new practices, which are critical to successful implementation efforts.

#### Provider morale

Another critical indicator of successful EBP implementation is provider morale. In Viteles’ [14] conceptualization, morale is comprised of job satisfaction and organizational commitment, an approach that has been adopted in more recent research [15]. In addition to being an important outcome in its own right, morale is a primary contributor to staff turnover, performance outcomes, and EBP use [16,17]. Initial evidence targets worker morale a potential key barrier to the successful implementation of new EBPs [17]. It is plausible that worker morale may not necessarily influence the adoption of innovations, but instead impact the quality or fidelity with which the EBP is delivered or implemented, although more research is needed in this area. Given the importance of retaining a productive and high-performing workforce in realizing successful implementation efforts [18], substance abuse treatment agencies are pressed to consider ways to bolster provider morale. Together, organizational learning climate and provider morale reflect the capacity of substance abuse treatment agencies to successfully adopt, implement, and sustainably use evidence-based interventions in practice. Given existing knowledge on organizational climate and employee morale, it is likely that these important outcomes are largely determined by individual characteristics, organizational factors, and the interactions or transactions between these phenomena.

### Key individual and organizational characteristics

#### Evidence-based practice attitudes

Among the most commonly studied provider-level characteristics in health services research is that of attitudes towards the adoption and use of evidence-based practices [19-21]. These attitudes are comprised of several facets, including one’s openness toward trying new interventions, perceived importance of empirical evidence relative to personal experience, intuitive appeal of EBPs, and the willingness to adopt EBPs if mandated by one’s institution [19]. Health care practitioners vary greatly in their attitudes toward EBP implementation, and this can have important implications for adopting and using EBPs with fidelity [22,23]. When substance abuse providers are open to new EBPs and are prepared for change, innovative and scientifically validated practices are more likely to be utilized [9,24-26]. However, while demonstrated to be critical to implementation success, EBP attitudes only partially explain provider- and organization-level outcomes. As provider attitudes do not exist within a vacuum, it is important to also consider how they interact with agency characteristics.

#### Agency expectations for EBP use

An essential organization-level counterpoint to provider attitudes is the degree to which agencies expect their workers to utilize EBPs with clients. Such expectations show a clear organizational commitment to and emphasis on adoption and use of EBPs. Substance abuse treatment centers that prioritize goals of EBP implementation by providing ongoing coaching and monitoring to providers are better able to fully implement EBPs and sustain them over time [5,26-28]. However, despite the advantages of clear agency prioritizations, direct service providers may respond differently to these expectations based on their personal attitudes toward EBPs, suggesting a need for alignment between agency and provider priorities.

#### Agency resources for EBP use

While distinct from expecting providers to use EBPs, an agency’s available resources to facilitate providers’ use of new and empirically-supported interventions also indicates an agency’s commitment to the provision of EBPs to clients. The presence of these resources does not necessarily communicate an explicit expectation for providers to adopt such practices. Instead, resources facilitate EBP use by providing supervision support, appropriate referral resources, necessary human capital, and adequate time and space to conduct new interventions. Similar to agency expectations, however, these resources may only have positive implications for worker and agency outcomes to the extent that they are met with favorable EBP attitudes in providers [29].

### Theoretical framework

Most research to date has focused on either individual-level (e.g., personality variables) or organization-level (e.g., job characteristics) factors that determine work-related outcomes such as organizational climate and employee morale [13,30,31]. More comprehensive approaches tend to use multiple units of analysis and strategies to account for the transactional nature of individual-level and organizational-level characteristics. However, far less empirical attention has been given to interactionist or transactional models of organizational phenomena, such as Person-environment fit (P-E fit) theory, despite well-established frameworks to guide such investigation. P-E fit is associated with a variety of individual and organizational level outcomes and is grounded in a rich history of theory and empirical research, particularly in organizational psychology and related fields [32-34]. The most commonly studied area of this framework is Person-organization fit (P-O fit), which theorizes that as fundamental characteristics at the provider level (e.g. values and attitudes) and at the organizational level (e.g. expectations of providers and resources) become more closely matched, outcomes such as satisfaction, well-being, work processes, and performance improve [35,36]. That is, compatibility between the values, attitudes, needs, abilities, resources, and other characteristics of the organization and members are critically important to indicators of success at both levels. Specifically, this fit (or lack thereof) may be characterized by the degree to which a provider’s attitudes toward a given work-related practice (e.g., evidence-based practices) are matched by the agency’s clear investment in and commitment to implementing such practices.

Substance abuse treatment agencies are increasingly being pressured to adopt EBPs through funding initiatives, leading to heightened agency expectations to use EBPs. However, clinicians may be less inclined to change their already existing practices if they were not properly convinced of the usefulness of these innovations [5]. P-O fit theory suggests that this discrepancy may be detrimental to outcomes such as organizational learning climate and provider morale. The review by Miller et al. [5] also argues that many organizations do not designate enough resources (e.g. training, mentorship, protected time) to properly support the implementation of EBPs, which may be particularly frustrating to direct service providers who value and are committed to using these practices.

### Current study

The current study uses a P-O fit framework to examine the above situations, which are characterized by mismatching provider and agency priorities. Despite the rich history of P-O fit theory in organizational psychology, research on the implications of P-O fit in the health service field is far less common and, to our knowledge, no research has examined P-O fit within the context of substance abuse treatment agencies. We believe that viewing organizational phenomena through a transactional lens (i.e., P-O fit), rather than examining provider or agency factors alone, yields a greater understanding of the determinants of EBP implementation in substance abuse treatment agencies.

As such, the present study focuses on the extent to which substance abuse providers’ *attitudes* toward evidence-based practices fit with agencies’ investment in and commitment to these practices, namely 1) *expectations* to use and 2) *resources* supporting the use of evidence-based practices. Prior research suggests that discrepancies between levels (i.e., P-O misfit) may have important implications for both individuals and organizations. The current research explores the influence of P-O fit on *provider morale* and perceived *organizational learning climate*, which are outcomes associated with successful EBP implementation. In light of this focus, we advance the following hypotheses:

*Hypothesis 1a: Discrepancies between provider-level EBP attitudes and agency-level expectations will negatively predict provider morale.*

*Hypothesis 1b: Discrepancies between provider-level EBP attitudes and agency-level resources will negatively predict provider morale.*

*Hypothesis 2a: Discrepancies between provider-level EBP attitudes and agency-level expectations will negatively predict organizational learning climate.*

*Hypothesis 2b: Discrepancies between provider-level EBP attitudes and agency-level resources will negatively predict organizational learning climate.*

## Methods

### Sample

Participants represented a convenience sample of 120 direct service providers from four separate community-based substance abuse treatment agencies in a large Midwestern U.S. city. The number of respondents per agency ranged from 6 to 55 service providers (i.e., 6, 10, 49, and 55). This distribution reflected the varying sizes of each agency’s workforce, as at least 80% of direct service providers from each agency responded to the survey. Two of the agencies represented outpatient substance abuse treatment centers, and two of the agencies represented agencies with inpatient, outpatient, and residential treatment for drug dependency. None of the agencies were located in hospital settings, but all had joined a community-academic partnership approximately one year prior and therefore had a similar history of collaborating with academic researchers. Table 1 summarizes the sample characteristics. Slightly more than half of respondents worked in outpatient settings, with the remainder working in inpatient settings and addiction recovery settings, providing services such as case management, housing, and employment support. The sample was predominately Caucasian and female, with an average age of 43. On average, participants had worked in the addiction services profession for eight years and within their current positions for just over four years. More than three-quarters of participating staff had earned a bachelor’s degree or higher, and more than half had been trained in social work, psychology, or counseling fields.

**Table 1:** **Sample demographics (N = 120)**

**Variables**	**Description**	**%**	**Mean (SD)**	**Range**
Gender	Female	65		
Race/Ethnicity	Caucasian	68		
Level of Education	High School	8		
	Associates	13		
	Bachelors	38		
	Masters and higher	41		
Position	Frontline provider	85		
	Supervisor-level provider	15		
Field of Study	Psychology/Counseling	35		
	General	24		
	Social Work	19		
	None (High School)	9		
	Education/Business/Public	8		
	Nursing/Allied Health	6		
Work Setting	Inpatient/Detox	8		
	Outpatient	38		
	Long-term Residential	13		
	Other Support Services	42		
Age			43.04(12.09)	22 – 73
Years in present job			4.04 (4.32)	0 – 27
Years in addiction services			8.02(8.02)	0 – 34
EBPAS Score			2.79(0.47)	1.07 – 3.80
Agency Expectations			2.41(1.04)	0.00 – 4.00
Agency Resources			2.69 (0.67)	0.44 – 4.00
Provider Morale			2.89 (0.78)	0.88 – 4.00
Learning Climate			2.40 (0.84)	0.00 – 3.80

*Note*. The following variables have missing observations: Years in addiction services (n = 1), position, EBPAS score and Agency Resources (n = 2), field of study and years in present job (n = 3), age (n = 4), race (n = 6), Gender (n = 7) and agency expectations (n = 10).

### Procedure

Participants were identified and recruited through administrative leaders at each of the four agencies. Administrative leaders notified their respective service providers that researchers would be visiting each agency and endorsed the study though agency-wide communications which facilitated voluntary participation from interested providers. Paper-and-pencil surveys were administered in-person during staff meetings by research team members who traveled to each participating agency. Prior to survey administration, participants were informed that, by evidence-based practices, we were referring to empirically-supported manualized therapies, treatments, and interventions with specific guidelines or components outlined in a manual that are to be followed in a structured or predetermined way. Data collection occurred in group settings; however, participants completed the surveys individually with no agency administrators present. Study procedures, including ensuring informed consent, were approved by Washington University’s Human Research Protection Office. Within each agency, the research team reached a response rate of at least 80% which served to minimize sampling bias.

### Measures

#### Dependent variables

##### *Organizational learning climate*

Based on the conceptualization of organizational learning climate from prior research [11-13], a 15-item measure was developed to assess the motivation and capacity of the organization to learn and incorporate new practices. The measure collected responses related to substance abuse agencies’ motivation to learn, flexibility, psychological safety, communication styles, and performance improvement values. Sample items included “Our team leader(s) frames challenges in a way that motivates the team to learn” and “Our team leader(s) encourages innovation at work.” The 0-4 response scale (0 = *not at all*; 4 = *to a very great extent*) used for this measure yielded high internal consistency (α = .95).

##### *Provider morale*

In line with prior research on provider morale [15,37-39], an 8-item measure was developed to assess providers’ job satisfaction and commitment to their organization. Sample items included “I am very happy to be working for this organization” and “I know this is the best place for me to work.” Again, the 0-4 response scale (0 = *not at all*; 4 = *to a very great extent*) was used for this measure, and internal consistency was high (α = .89).

#### Independent variables

##### *Attitudes toward empirically supported treatments*

The Evidence-Based Practice Attitudes Scale (EBPAS) [19] was used to measure provider attitudes toward using new types of manualized therapies, interventions, or treatments. The EBPAS consisted of 15 Likert-type items scored on the following 0-4 response scale: *not at all*, *to a slight extent*, *to a moderate extent*, *to a great extent*, and *to a very great extent*. Per Aarons [19], an overall mean scale score was computed based on items from four subscales: intuitive *Appeal* of manualized practices; adoption likelihood given various *Requirements*; *Divergence* of usual practice from research-based therapies (reversed-scored); and *Openness* to learning and using new interventions and practices. The internal consistency coefficient of the total EBPAS scale in our sample was high (α = .80), which is comparable to previous studies [19,20,40]. Further, alpha coefficients for each subscale were as follows: Appeal (α = .79), Requirements (α = .92), Divergence (α = .67), and Openness (α = .86).

##### *Agency expectations for use of EBPs*

Staff members were asked the extent to which their agency expected its workers to “use scientifically proven practices”. The same 0-4 response scale as above (0 = *not at all*; 4 = *to a very great extent*) was used for this item.

##### *Agency resources for use of EBPs*

Respondents were asked a set of questions that tapped into the extent to which their agency possessed and provided resources necessary to support the implementation and use of new interventions. The measure consisted of eight items, including the following: “My agency provides the supports needed to implement any new intervention” and “My agency has a program manager/supervisor who can ensure the successful implementation of a new intervention.” The 0-4 response scale (0 = *not at all*; 4 = *to a very great extent*) was used for this measure. The internal consistency of this measure was high (α = .84).

### Analysis

First, all independent variables were centered and standardized [41]. Next, univariate and bivariate analyses were conducted with key dependent, independent, and demographic variables. Then, to model provider-agency fit, we computed difference scores, a widely used analytic approach to modeling P-E fit [33,42]. To calculate difference scores, agency resources and agency expectations were subtracted from provider EBP attitudes [EBPAS minus (-) Resources; EBPAS minus (-) Expectations], respectively. This computation yielded two separate provider-agency fit indicators, in which a score of zero represented perfect match between the provider and agency characteristics. Positive scores on these fit indicators represented cases in which provider attitudes were more favorable towards EBPs than agency variables. Negative scores represented cases in which agency variables were more favorable towards EBPs than provider attitudes.

In testing all hypotheses, quadratic regression analyses of this difference score were conducted to determine whether or not a curvilinear model of provider-agency discrepancies (i.e., divergence from a score of zero indicating perfect match) predicted organizational learning climate and provider morale. Quadratic regression reflects a second-order polynomial function that uses a parabolic (i.e., curvilinear) equation to best fit a given set of data [32]. While linear regression may represent a simpler analysis, this approach would only yield evidence regarding whether or not a specific *direction* of misfit predicted the dependent variables. Importantly, linear regression analyses would not be sensitive to patterns such as reverse U-shaped curves, for instance, in which both directions of misfit yield negative outcomes. That is, linear analyses would not detect provider-agency misfit if both directions of misfit (provider attitudes more favorable than agency characteristics, and agency characteristics more favorable than provider attitudes) were equally predictive of the dependent variables. Thus, while the linear models were included and accounted for in these analyses, it was determined that quadratic regression analyses would more adequately and comprehensively model the influence of provider-agency fit and misfit. In doing this, we were able to model the level of the dependent variables across the entire “fit continuum” (provider attitudes less favorable, equally favorable, and more favorable than agency characteristics).

## Results

### Descriptive and correlational statistics

Frequency and mean statistics are reported in Table 1 for both substantive and demographic variables. Data, including computed variables of fit, were normally distributed, and the demographics were largely representative of the broader service provider population. No significant differences in evidence-based practice attitudes were found between the four agencies. Pearson correlation coefficients between the majority of substantive and demographic variables are provided in Table 2. No significant differences were found between frontline and supervisor-level providers for any of the key study variables. EBPAS scores were negatively associated with years at one’s current job. Also, while distinct constructs, there was a moderate positive relationship between agency expectations and agency resources. Similarly, there was a moderately-to-highly positive association between learning climate and morale.

**Table 2:** **Intercorrelations between measured variables**

**Variable**	**1**	**2**	**3**	**4**	**5**	**6**	**7**	**8**	**9**	**10**	**11**
1. EBPAS	*--*	.06	.03	-.04	-.04	-.14	-.15	-.25**	-.11	.14	.00
2. Expectations		--	.48**	.37**	.36**	.15	.17	.04	.11	-.21*	.00
3. Resources			--	.64**	.62**	.16	.11	-.05	.14	-.24	.01
4. Learning Climate				--	.63**	.16	.02	.02	.10	-.25**	.07
5. Morale					--	.14	.22*	.14	.14	-.35**	.14
6. Gender						--	.22*	.15	.12	-.06	.12
7. Age							--	.44**	.62**	-.11	.28**
8. Years – job								--	.51**	.05	.23*
9. Years – profession									--	.09	.25**
10. Education level										*--*	.10
11. Position											--

*Note*. Gender (0 = Female; 1 = Male). Position (0 = Frontline Provider; 1 = Supervisor-level Provider) * *p* < .05. ** *p* < .01.

### Provider-agency (P-A) fit on provider morale

Linear regression analyses indicated that the difference score between Provider EBPAS and Agency Resources (EBPAS – Resources) significantly predicted lower provider morale, β = -.468, *t*(115) = -5.677, *p* < .001, *R*^2^ = .22. Even after accounting for the linear relationship, however, quadratic regression analyses indicated a significant curvilinear relationship involving the discrepancies between Provider EBPAS and Agency Resources and the outcome of provider morale, β = -.188, *t*(114) = -2.158, *p* = .033, *R*^2^ = .25, lending support for Hypothesis 1a.

Similarly, linear regression analyses indicated that the difference score between Provider EBPAS and Agency Expectations (EBPAS – Expectations) significantly predicted lower provider morale, although to a lesser degree than the influence of the discrepancy between attitudes and resources, β = -.277, *t*(107) = -2.984, *p* = .004, *R*^2^ = .08. Again, however, accounting for this linear association, quadratic regression analyses indicated a significant curvilinear relationship involving the discrepancies between Provider EBPAS and Agency Expectations and the outcome of provider morale, β = -.258, *t*(106) = -2.783, *p* = .006, *R*^2^ = .14, lending support for Hypothesis 1b.

**Figure 1:**
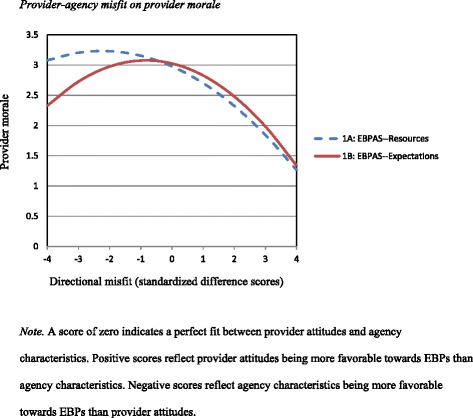
Provider-agency misfit on provider morale.

Figure 1 depicts the quadratic relationships of these data. This graph reflects the significant curvilinear, reverse U-shaped associations between 1) the discrepancies between provider EBP attitudes and agency resources on provider morale, and 2) the discrepancies between provider EBP attitudes and agency expectations on provider morale. For both P-A fit relationships, provider morale was highest roughly at the point of zero discrepancy (i.e., perfect fit) between provider and agency variables. Relatedly, for both associations, provider morale declined as the discrepancies between provider and agency variables increased. As illustrated in Curve 1a, this was particularly true for cases of positive discrepancy, which were characterized by provider EBP attitudes being more positive than agency resources.

### Provider-agency (P-A) fit on organizational learning climate

Linear regression analyses indicated that the difference score between Provider EBPAS and Agency Resources (EBPAS – Resources) significantly predicted lower organizational learning climate, β = -.478, *t*(115) = -5.844, *p* < .001, *R*^2^ = .23. Accounting for this linear relationship, however, quadratic regression analyses indicated a significant curvilinear relationship involving the discrepancies between Provider EBPAS and Agency Resources and the outcome of organizational learning climate, β = -.293, *t*(114) = -3.504, *p* = .001, *R*^2^ = .30, lending support for Hypothesis 2a.

Likewise, linear regression analyses indicated that the difference score between Provider EBPAS and Agency Expectations (EBPAS – Expectations) significantly predicted lower organizational learning climate, although again to a lesser degree than the influence of the discrepancy between attitudes and resources, β = -.286, *t*(107) = -3.092, *p* = .003, *R*^2^ = .08. Even after accounting for this linear association, however, quadratic regression analyses again indicated a significant curvilinear relationship involving the discrepancies between Provider EBPAS and Agency Expectations and the outcome of organizational learning climate, β = -.225, *t*(106) = -2.420, *p* = .017, *R*^2^ = .13, lending support for Hypothesis 2b.

**Figure 2:**
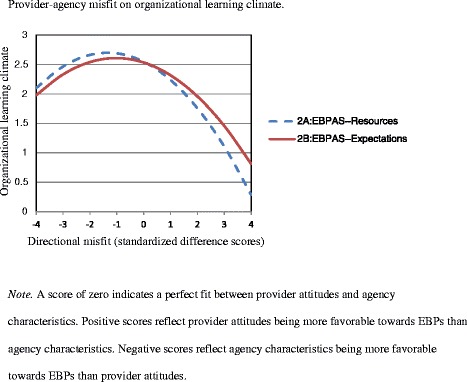
Provider-agency misfit on organizational learning climate.

Figure 2 illustrates the quadratic relationships of these data. This graph reflects the significant curvilinear, reverse U-shaped associations between 1) the discrepancies between provider EBP attitudes and agency resources on organizational learning climate, and 2) the discrepancies between provider EBP attitudes and agency expectations on organizational learning climate. Similar to findings for provider morale, organizational learning climate was highest roughly at the point of zero discrepancy (i.e., perfect fit) between provider and agency variables (both Expectations and Resources). Relatedly, for both associations, organizational learning climate declined as the discrepancies between provider and agency variables increased. As depicted in Curve 2a, this was particularly true for cases of positive discrepancy, which were characterized by provider EBP attitudes being more positive than agency resources.

## Discussion

Results of this study consistently indicated that misalignment between provider and agency characteristics, with regard to implementing EBPs, was an indicator of poor provider morale and weak organizational learning climate in selected substance abuse treatment agencies. Generally speaking, highest provider morale and strongest organizational learning climates were reported by providers who had EBP attitudes that matched the degree to which their agencies provided expectations and resources supportive of EBP use. When provider attitudes and perceptions of agency expectations and resources were less aligned, however, these provider- and organization-level outcomes suffered. These findings yield additional support for P-O fit theory, indicating that morale and climate are dependent on the congruence between provider-level attitudes towards using EBPs and agency-level expectations and resources that indicate prioritization of EBP implementation.

### Direction of discrepancy

While the focus of this study was to examine the implications of provider-agency discrepancies, one might raise the question of which *direction* of discrepancy more strongly predicts negative outcomes (i.e., provider morale and organizational learning climate). Interestingly, all four quadratic analyses indicated that outcomes (both provider morale and organizational learning climate) were at a more negative level (i.e., y-intercept) and more strongly predicted in the negative direction (i.e., slope) when provider attitudes were more favorable towards EBPs than agency resources and expectations (see Figures 1 and 2). This is corroborated by the linear analyses, all of which were significant in the negative direction. Misfit characterized by provider attitudes being more favorable towards EBPs than agency characteristics predicted worse outcomes than misfit in which provider attitudes were less favorable towards EBP use than agency characteristics.

This was particularly true for agency resources; provider morale and organizational learning climate were especially low when provider attitudes toward EBPs were more favorable than the agency resources necessary to successfully implement EBPs. That is, outcomes were most negative when providers viewed EBPs favorably, but perceived their agencies’ expectations for EBP use to be relatively low and agencies’ resources necessary for EBP implementation to be relatively limited. This suggests that provider morale and organizational learning climate suffer most when providers who value and desire to implement EBPs in their addictions practice do not perceive an adequate amount of support, time, supervision, or other organizational resources to effectively conduct these practices.

Relatedly, our findings suggest that having a slight abundance of organizational resources and supports, relative to one’s own EBP attitudes, may not be very harmful to provider morale and organizational learning climate. This situation does not explicitly require providers to use EBPs more than desired or sacrifice personal control over their work environment. However, it seems that the abundance of agency expectations to use EBPs, relative to providers’ EBP attitudes, may be slightly more detrimental for outcomes, particularly provider morale. This finding aligns with a great deal of organizational literature which has established that employees who are not given control in their work environment, especially when they prefer such control, are more stressed and perform worse [43,44]. For direct service providers who are less open to EBPs, high agency expectations to use EBPs may increase workers’ perceived stress levels by decreasing their sense of personal control over their work environment. In turn, this loss of perceived work control may compromise these providers’ job satisfaction, increase job strain, and damage morale.

### Implications for health services practice

This study benefits from being heavily grounded in a strong theoretical framework that has been highly useful in organizational research for decades. Importantly though, these findings also have substantial practical implications for substance abuse treatment agencies and for implementation science. For instance, researchers and agency leaders may find utility in employing targeted implementation strategies, as appropriate [45]. Specifically, conducting local needs assessments, identifying organizational and employee-level barriers, conducting local consensus discussions, and tailoring strategies to overcome barriers and honor preferences likely represent key implementation activities that acknowledge the importance of fit between providers and inner setting dimensions of a complex implementation process.

The results suggest that, at a minimum, managers and supervisors should periodically conduct self-assessments of the agency’s cultural predispositions toward EBP implementation (e.g., communicated expectations, supportive resources, technical assistance, etc.) and providers’ openness, abilities, perceived value, acceptability, and general attitudes towards using EBPs [46]. Identifying disconnects between these agency and provider antecedents to use of EBPs may elicit critical awareness of implementation barriers. This knowledge may also pinpoint strategies that can be used to bring provider and agency values, priorities, and attitudes more closely aligned to ease the process of implementing EBPs in substance abuse treatment agencies. For instance, a relative deficiency in one facet of provider attitudes, such as openness towards using EBPs, may underscore the need for initiating an organizational campaign focused on improving provider awareness of empirically-supported EBPs, providing sufficient training opportunities, and modeling the successful use of EBPs in small-scale, trial formats. Conversely, identifying a deficiency in agency resources supportive of EBP implementation, relative to provider attitudes, may highlight a need to prioritize financial and human capital resources specifically toward supporting the successful implementation and use of EBPs.

### Limitations

Findings from this study should be interpreted with caution for the following reasons. For one, we utilized a cross-sectional design which limited our ability to make causal inferences. It is possible that lower provider morale and weaker organizational learning climates may pull provider and agency characteristics further apart over time. However, P-O fit theory suggests the reverse explanation—that poorly fitting employee and organizational characteristics weaken outcomes such as morale and climate. As another potential limitation, the current measures of fit represented computations of self-reported provider and agency variables. It was not a direct measure of perceived fit, which is an approach that has demonstrated predictive validity in some research [47]. However, indirect measures of fit, such as the one used in this study, avoid confounding the independent effects of the person and environment with their combined effect [48]. Further, the indirect assessment of provider-agency fit was a more covert measure of the primary predictor variable, thereby reducing the confounding influence of demand characteristics which are common in self-reported data.

Certainly, there are potential applications of person-organization fit along a number of other factors not measured in the current study (e.g., salary, mission, job characteristics), as well as alignment on any particular EBP. While the current study was focused on broader attitudes and antecedents toward EBPs in general, study of more specific factors, perceptions, and interventions is likely a fruitful area for future research. Future research should also examine the impact of person-organization fit on outcomes that are more proximal to actual implementation processes, such as acceptability and fidelity. We also acknowledge the potential limitations to our use of a one-item measure of Agency Expectations, but note the relative lack of available and validated measures of this construct within the current context.

It could also be argued that negative EBP attitudes in providers coupled with either low EBP expectations or resources in agencies, a scenario reflecting person-agency fit, should not predict strong organizational learning climate. While the weakest organizational learning climates were found in cases of provider-agency misfit, as hypothesized, the current data suggest that organizational learning climates were stronger in “high-high” scenarios (i.e., positive provider attitudes and high agency expectations/resources) than in “low-low” scenarios (i.e., negative provider attitudes and low agency expectations/resources). In the current sample, more respondents could be classified by the “high-high” scenario (*n* = 37 with above average attitudes and expectations; *n* = 39 with above average attitudes and resources) than by the “low-low” scenario (*n* = 21 with below average attitudes and expectations; *n* = 25 with below average attitudes and resources). Therefore, the influence of provider-agency fit on organizational learning climate may have been most strongly driven by the “high-high” type of provider-agency fit.

Finally, analysis of multiple organizations often warrants multilevel modeling to account for within-organization clustering of data. However, in the current study, within-organization variances among the key independent and dependent variables were not systematically different than variances in the overall sample, suggesting minimal clustering. Further, sensitivity analyses indicated similar within-organization relationships among variables to those reported in the current results, yielding confidence in the statistical approach and robustness of the findings.

## Conclusions

The current research benefits from a strong theoretical framework, consistent findings in line with a priori hypotheses, and significant practical implications for substance abuse treatment agencies. Provider morale and organizational learning climates are important indicators of successful EBP implementation. Results of this study indicate that a comprehensive attempt to strengthen these outcomes must consider both provider- and agency-level characteristics regarding EBP implementation. Organizational efforts to more closely align provider attitudes and agency priorities, such as through local consensus discussions to gain buy-in among diverse stakeholders, will likely constitute a key strategy in fostering the implementation of EBPs in substance abuse treatment organizations.

## Abbreviations

EBP: Evidence-based practice
EBPAS: Evidence-Based Practice Attitude Scale
P-E Fit: Person-environment fit
P-O fit: Person-organization fit
P-A fit: Provider-agency fit
